# Supplementary material for: A Field Efficacy Trial of Recombinant Porcine Circovirus Type 2d Vaccine in Three Herds
Source: Vaccines (Basel). 2023 Sep 16;11(9):1497. doi: 10.3390/vaccines11091497 (PMC10538009; doi:10.3390/vaccines11091497)
Supplement: Supplementary file 1 [file vaccines-11-01497-s001.zip › vaccines-2551420-supplementary.pdf]

Supplementary data S1. Serum IgG level of piglets.

Commercial PCV2 ELISA(PCV2a-based)

		farm H						farm M						farm Y					
		3	7	11	15	19	23	3	7	11	15	19	23	3	7	11	15	19	23
groups	number	0wpv	4wpv	8wpv	12wpv	16wpv	20wpv	0wpv	4wpv	8wpv	12wpv	16wpv	20wpv	0wpv	4wpv	8wpv	12wpv	16wpv	20wpv
	1	0.293	0.554	0.562	0.502	0.447	1.092	1.997	1.431	0.558	0.650	2.147	1.946	0.587	0.208	0.217	0.315	0.392	1.534
	2	0.609	1.129	0.977	0.937	0.795	1.190	1.889	1.281	0.366	0.364	1.419	1.648	1.012	0.360	0.190	0.553	0.517	1.026
	3	0.440	0.138	0.140	0.256	0.156	0.245	1.977	1.565	0.681	0.572	1.525	1.691	0.452	0.116	0.111	0.389	0.400	1.201
	4	0.303	0.168	0.560	0.609	1.429	1.466	1.285	0.449	0.164	0.416	1.613	1.722	0.461	0.143	0.106	0.078	0.276	1.058
	5	0.486	0.225	0.322	0.284	0.785	0.902	0.384	0.440	0.461	0.530	1.420	1.555	0.909	0.288	0.148	0.382	0.829	1.224
	6	0.492	0.171	0.384	0.585	1.101	0.999	0.267	1.219	0.485	0.530	1.484	1.604	0.949	0.498	0.529	0.469	1.621	1.644
	7	0.525	0.179	0.385	0.424	0.200	0.463	1.869	1.380	0.610	0.480	1.584	1.713	0.806	0.243	0.265	0.271	1.028	1.157
vaccine	8	0.309	0.360	0.482	0.313	0.345	0.771	1.662	1.033	0.644	0.632	1.628	1.732	0.424	0.242	0.293	0.312	0.591	1.181
	9	0.251	0.187	0.481	0.883	1.459	1.556	1.742	1.280	0.498	0.474	1.631	1.558	0.539	0.213	0.216	0.192	0.311	1.562
	10	0.171	0.426	0.745	0.768	1.325	1.564	1.433	0.851	0.663	1.270	1.715	1.475	0.884	0.366	0.281	0.222	0.364	1.542
	11	0.124	0.483	0.178	0.680	1.382	1.328	1.513	0.687	0.355	1.181	1.620	1.773	0.520	0.832	0.479	0.389	0.477	0.396
	12	0.353	0.238	0.406	0.288	0.824	1.197	1.554	0.742	0.487	0.917	1.724	1.581	0.382	0.148	0.203	0.367	0.437	0.881
	13	0.354	1.019	0.796	0.846	0.709	0.681	1.591	0.793	0.252	1.182	1.649	1.415	0.655	0.236	0.198	0.302	0.378	0.272
	14	0.353	0.203	0.637	0.623	1.638	1.491	1.530	0.983	0.602	0.834	1.786	1.558	0.900	0.310	0.169	0.124	1.484	1.663
	15	0.383	0.180	0.301	0.238	1.312	1.303	0.918	0.432	0.275	0.398	0.678	1.636	0.627	0.186	0.235	0.302	1.072	1.456
	16	0.056	0.013	0.469	0.963	1.668	1.731	1.646	0.771	0.126	1.327	1.702	1.874	0.719	0.150	0.026	0.010	0.582	1.541
	17	0.458	0.103	0.313	0.762	1.644	1.732	2.097	2.224	1.244	1.386	1.655	1.695	0.532	0.150	0.034	0.010	1.168	1.663
control	18	0.043	0.013	0.141	0.745	1.734	1.677	0.702	0.220	0.041	0.036	1.847	1.765	0.459	0.128	0.033	0.447	1.491	1.634
	19	0.055	0.014	0.416	0.822	1.573	1.545	1.798	1.369	0.309	0.147	1.558	1.743	1.463	0.691	0.133	0.034	0.643	1.330
	20	0.067	0.019	0.323	0.712	1.665	1.743	2.099	2.232	1.236	0.916	1.581	1.701	1.418	0.592	0.129	0.025	1.490	1.650
vaccine	ave	0.363	0.377	0.490	0.549	0.927	1.083	1.441	0.971	0.473	0.695	1.575	1.641	0.674	0.293	0.243	0.311	0.678	1.187
	geomean	0.337	0.297	0.436	0.496	0.759	0.982	1.279	0.893	0.442	0.640	1.538	1.636	0.641	0.257	0.220	0.282	0.577	1.077
control	ave	0.136	0.032	0.332	0.801	1.657	1.685	1.668	1.363	0.591	0.762	1.669	1.756	0.918	0.342	0.071	0.105	1.075	1.564
	geomean	0.083	0.021	0.308	0.796	1.656	1.684	1.557	1.029	0.300	0.388	1.666	1.754	0.817	0.260	0.055	0.033	0.994	1.558

in-house ELISA(PCV2d-based)

		farm H						farm M						farm Y					
		3	7	11	15	19	23	3	7	11	15	19	23	3	7	11	15	19	23
groups	number	0wpv	4wpv	8wpv	12wpv	16wpv	20wpv	0wpv	4wpv	8wpv	12wpv	16wpv	20wpv	0wpv	4wpv	8wpv	12wpv	16wpv	20wpv
	1	0.175	1.186	2.355	1.181	0.771	1.950	1.651	0.626	1.638	1.102	3.368	3.039	0.787	1.076	1.953	1.318	1.224	3.151
	2	0.304	2.098	3.037	1.878	1.374	2.139	1.351	1.267	2.671	1.362	2.271	2.870	0.704	0.801	1.763	1.801	1.505	2.013
	3	0.232	0.954	2.369	1.581	0.996	1.165	2.678	1.752	2.528	1.568	2.489	3.114	0.584	0.585	1.195	1.257	1.062	2.282
	4	0.155	1.582	2.687	1.586	2.665	2.517	0.435	1.486	2.688	1.634	2.473	3.106	0.532	0.581	1.117	0.451	1.027	2.169
	5	0.252	1.024	2.249	1.023	2.031	1.793	0.244	2.302	2.902	1.749	2.199	2.773	0.623	2.238	2.456	1.475	2.433	3.018
	6	0.256	1.650	2.331	1.465	2.178	1.740	0.110	1.447	2.508	1.209	2.060	2.595	0.654	0.803	1.931	0.954	3.138	3.329
	7	0.270	1.543	2.396	1.218	0.670	0.882	1.479	0.743	1.420	0.791	2.303	2.766	0.543	0.508	1.784	1.252	1.736	1.776
vaccine	8	0.150	1.380	2.458	0.946	0.899	1.905	2.054	0.968	1.961	1.116	2.444	2.337	0.250	0.830	1.689	1.041	1.251	2.020
	9	0.127	1.506	2.602	2.152	2.768	2.960	1.732	1.461	2.458	1.348	2.900	2.774	0.495	0.689	1.341	1.034	0.971	3.094
	10	0.114	2.489	3.026	1.897	2.683	3.242	1.392	0.665	1.818	0.668	3.038	3.129	0.689	1.464	2.116	1.300	1.221	2.995
	11	0.085	1.075	1.923	1.770	2.568	2.372	1.104	2.145	2.884	3.193	2.459	3.330	0.311	0.819	1.969	1.341	0.913	0.595
	12	0.177	1.111	2.596	1.026	1.697	2.243	0.756	1.943	2.892	2.634	3.188	2.951	0.198	0.948	1.811	1.611	1.315	1.617
	13	0.151	1.703	2.637	1.893	1.128	0.933	1.475	1.491	1.825	0.677	3.220	2.880	0.299	0.968	2.391	1.865	1.246	0.698
	14	0.155	1.141	2.668	1.551	2.951	2.740	0.573	1.988	2.806	2.122	3.256	3.011	0.711	0.860	1.560	0.802	2.854	3.013
	15	0.182	1.794	2.816	1.390	2.923	2.754	0.874	1.856	2.495	1.594	0.349	3.010	0.342	0.993	1.835	1.376	2.000	2.740
	16	0.067	0.112	2.243	3.204	3.335	3.358	0.631	0.212	0.242	3.279	2.465	3.148	0.450	0.196	0.172	0.145	3.358	3.313
	17	0.184	0.156	1.397	3.131	3.351	3.397	3.059	2.942	2.434	2.055	2.464	2.998	0.446	0.204	0.170	0.104	3.207	3.284
control	18	0.063	0.120	1.735	3.126	3.358	3.340	0.655	0.296	0.309	0.194	3.300	3.295	0.284	0.169	0.151	3.050	3.364	3.312
	19	0.066	0.110	1.799	3.153	3.309	3.301	1.907	0.867	0.389	0.155	2.768	3.264	1.426	0.580	0.332	0.126	3.038	3.253
	20	0.070	0.110	1.919	3.110	3.277	3.267	3.084	2.744	2.195	0.965	2.422	2.441	1.408	0.535	0.313	0.166	3.242	3.338
vaccine	ave	0.186	1.482	2.543	1.504	1.887	2.089	1.194	1.476	2.366	1.518	2.535	2.912	0.515	0.944	1.794	1.258	1.593	2.301
	geomean	0.176	1.429	2.527	1.459	1.678	1.954	0.926	1.368	2.311	1.379	2.320	2.902	0.476	0.879	1.753	1.196	1.474	2.078
control	ave	0.090	0.122	1.818	3.145	3.326	3.333	1.867	1.412	1.114	1.330	2.684	3.029	0.803	0.337	0.227	0.718	3.242	3.300
	geomean	0.081	0.120	1.797	3.145	3.326	3.332	1.494	0.848	0.690	0.722	2.665	3.012	0.648	0.291	0.215	0.249	3.239	3.300

Supplementary data S2. NA titers.

		vaccine															control					vaccine		control	
		1	2	3	4	5	6	7	8	9	10	11	12	13	14	15	16	17	18	19	20	mean	sd	mean	sd
farm H	0wpv	8	8	8	8	8	8	8	8	8	8	8	8	8	8	8	8	8	8	8	8	8.00	0.00	8.00	0.00
	4wpv	64	128	32	32	64	64	64	64	128	256	64	64	256	128	256	8	8	8	8	8	110.93	81.01	8.00	0.00
	8wpv	128	256	128	64	64	128	128	128	128	512	64	256	64	64	64	8	8	8	8	8	145.07	119.65	8.00	0.00
	12wpv	64	128	128	128	64	128	64	64	128	128	128	64	256	128	128	2048	256	512	128	1024	115.20	49.57	793.60	780.70
	16wpv	32	128	64	512	256	256	32	64	512	256	256	256	64	512	512	2048	1024	512	2048	2048	247.47	186.77	1536.00	724.08
	20wpv	256	256	32	256	256	16	64	256	1024	256	256	512	64	256	256	2048	2048	2048	2048	2048	267.73	244.19	2048.00	0.00

		vaccine															control					vaccine		control	
		1	2	3	4	5	6	7	8	9	10	11	12	13	14	15	16	17	18	19	20	mean	sd	mean	sd
farm M	0wpv	8	8	8	8	8	8	16	32	16	8	8	16	8	8	8	8	8	8	8	16	11.20	6.62	9.60	3.58
	4wpv	64	64	64	64	64	128	16	128	128	64	128	128	128	64	128	16	256	16	64	256	90.67	38.09	121.60	124.24
	8wpv	64	256	64	128	256	32	16	64	128	64	128	128	128	512	256	8	128	8	8	8	148.27	127.78	32.00	53.67
	12wpv	32	128	128	128	128	64	8	128	128	64	1024	512	16	128	128	2048	512	8	8	8	182.93	260.41	516.80	883.35
	16wpv	1024	512	2048	2048	512	512	512	256	512	1024	512	1024	1024	1024	8	2048	2048	2048	256	2048	836.80	583.49	1689.60	801.41
	20wpv	2048	1024	2048	2048	1024	512	1024	256	256	256	2048	1024	512	1024	256	2048	512	2048	2048	2048	1024.00	711.03	1740.80	686.92

		vaccine															control					vaccine		control	
		1	2	3	4	5	6	7	8	9	10	11	12	13	14	15	16	17	18	19	20	mean	sd	mean	sd
farm Y	0wpv	8	8	8	8	8	8	8	8	8	8	8	8	8	8	8	8	8	8	8	8	8.00	0.00	8.00	0.00
	4wpv	16	16	32	16	128	32	32	32	32	128	256	16	64	16	16	8	8	8	32	32	55.47	67.03	17.60	13.15
	8wpv	64	128	64	64	128	64	64	64	64	128	128	128	64	32	32	8	8	8	8	8	81.07	36.01	8.00	0.00
	12wpv	64	128	128	32	64	64	64	64	64	64	128	128	128	32	64	8	8	1024	8	8	81.07	36.01	211.20	454.37
	16wpv	128	128	128	128	128	512	128	128	64	64	128	128	128	512	256	2048	512	2048	256	128	179.20	141.46	998.40	968.07
	20wpv	512	256	256	128	512	2048	256	256	512	1024	32	128	64	512	256	2048	2048	1024	2048	2048	450.13	508.34	1843.20	457.95

Supplementary data S3. Viremia (Ct value).

		vaccine															control					vaccine		control	
		1	2	3	4	5	6	7	8	9	10	11	12	13	14	15	16	17	18	19	20	mean	sd	mean	sd
farm H	0wpv	35.00	35.00	35.00	35.00	35.00	35.00	35.00	35.00	35.00	35.00	35.00	35.00	35.00	35.00	35.00	35.00	35.00	35.00	35.00	31.03	35.00	0.00	34.21	1.78
	4wpv	35.00	35.00	35.00	35.00	35.00	35.00	35.00	35.00	35.00	35.00	35.00	35.00	35.00	35.00	35.00	35.00	35.00	35.00	35.00	35.00	35.00	0.00	35.00	0.00
	8wpv	35.00	35.00	35.00	35.00	34.72	34.80	35.00	35.00	35.00	35.00	35.00	35.00	35.00	35.00	35.00	23.70	30.67	28.59	25.72	30.02	34.97	0.09	27.74	2.95
	12wpv	35.00	35.00	31.60	33.00	35.00	34.08	34.42	35.00	35.00	35.00	35.00	35.00	31.65	35.00	35.00	19.88	27.12	25.21	22.21	27.07	34.32	1.23	24.30	3.18
	16wpv	35.00	35.00	35.00	32.99	34.69	34.36	35.00	34.65	33.93	34.09	35.00	35.00	35.00	31.70	35.00	27.91	30.86	20.89	26.29	30.59	34.43	0.95	27.31	4.06
	20wpv	35.00	35.00	35.00	35.00	35.00	35.00	35.00	35.00	35.00	35.00	35.00	35.00	35.00	35.00	35.00	30.83	32.73	30.81	25.72	32.41	35.00	0.00	30.50	2.82

		vaccine															control					vaccine		control	
		1	2	3	4	5	6	7	8	9	10	11	12	13	14	15	16	17	18	19	20	mean	sd	mean	sd
farm M	0wpv	32.43	35.00	35.00	35.00	35.00	35.00	35.00	35.00	34.44	35.00	35.00	35.00	35.00	35.00	27.80	35.00	35.00	35.00	35.00	35.00	34.31	1.92	35.00	0.00
	4wpv	35.00	35.00	35.00	35.00	35.00	35.00	35.00	35.00	35.00	35.00	35.00	35.00	35.00	35.00	35.00	35.00	35.00	35.00	35.00	35.00	35.00	0.00	35.00	0.00
	8wpv	35.00	35.00	35.00	35.00	35.00	35.00	35.00	35.00	35.00	35.00	35.00	35.00	35.00	35.00	35.00	35.00	35.00	35.00	35.00	35.00	35.00	0.00	35.00	0.00
	12wpv	30.66	35.00	35.00	29.85	35.00	35.00	33.19	34.54	35.00	35.00	33.08	35.00	35.00	31.31	35.00	27.10	33.29	30.04	25.01	35.00	33.84	1.82	30.09	4.16
	16wpv	35.00	31.91	34.39	32.71	33.91	31.08	35.00	32.95	33.88	32.32	32.50	33.49	33.90	32.34	35.00	27.85	27.69	30.84	23.31	32.28	33.36	1.22	28.39	3.45
	20wpv	33.95	35.00	35.00	33.39	33.55	35.00	35.00	35.00	35.00	35.00	35.00	35.00	35.00	35.00	35.00	31.12	34.22	34.42	26.76	34.62	34.73	0.58	32.22	3.38

		vaccine															control					vaccine		control	
		1	2	3	4	5	6	7	8	9	10	11	12	13	14	15	16	17	18	19	20	mean	sd	mean	sd
farm Y	0wpv	35.00	35.00	35.00	35.00	35.00	35.00	35.00	35.00	35.00	35.00	35.00	35.00	35.00	35.00	35.00	35.00	35.00	35.00	35.00	35.00	35.00	0.00	35.00	0.00
	4wpv	35.00	35.00	35.00	35.00	35.00	35.00	35.00	35.00	35.00	35.00	35.00	35.00	35.00	35.00	35.00	35.00	35.00	35.00	35.00	35.00	35.00	0.00	35.00	0.00
	8wpv	35.00	35.00	35.00	35.00	35.00	35.00	35.00	35.00	35.00	35.00	35.00	35.00	35.00	35.00	35.00	35.00	35.00	35.00	35.00	35.00	35.00	0.00	35.00	0.00
	12wpv	31.70	33.83	35.00	30.15	34.20	35.00	35.00	29.01	35.00	34.77	35.00	35.00	33.25	35.00	35.00	35.00	35.00	29.34	33.64	33.18	33.79	1.96	33.23	2.32
	16wpv	29.32	31.22	35.00	35.00	35.00	31.64	33.50	35.00	29.41	34.41	33.95	31.45	27.75	34.58	25.82	27.03	23.73	24.52	25.86	25.46	32.20	2.99	25.32	1.27
	20wpv	35.00	35.00	35.00	35.00	35.00	35.00	35.00	35.00	35.00	35.00	35.00	35.00	35.00	35.00	34.18	32.20	29.78	32.23	29.72	25.88	34.95	0.21	29.96	2.59

Supplementary data S4. Body weight.

**body weight(unit; Kg)**		**farm H**				**farm M**				**farm Y**			
		**3**	**7**	**11**	**15**	**3**	**7**	**11**	**15**	**3**	**7**	**11**	**15**
groups	number	**0wpv**	**4wpv**	**8wpv**	**12wpv**	**0wpv**	**4wpv**	**8wpv**	**12wpv**	**0wpv**	**4wpv**	**8wpv**	**12wpv**
**vaccine**	1	8.8	15.4	28.8	41.3	8.0	14.5	32.8	56.2	7.6	19.5	33.1	48.2
	2	8.9	22.2	36.8	43.9	8.2	14.4	30.4	45.9	7.0	15.6	26.3	48.0
	3	6.6	17.3	35.2	56.5	7.6	19.1	30.9	49.8	7.3	19.9	27.6	52.8
	4	7.7	22.2	26.4	57.1	6.2	17.0	29.6	42.8	7.4	19.0	31.6	42.8
	5	7.0	16.0	30.6	39.9	6.5	15.8	29.3	45.6	7.3	17.3	26.0	44.7
	6	7.3	20.1	37.0	54.0	7.7	18.8	26.9	46.8	8.1	18.5	31.7	48.5
	7	6.6	17.5	29.8	47.3	7.6	15.7	35.5	45.5	7.0	19.5	29.5	41.1
	8	7.6	15.7	27.5	47.9	6.7	13.0	32.3	57.0	6.5	18.5	28.4	41.5
	9	6.2	16.5	25.2	42.3	6.1	16.3	31.0	47.9	7.6	16.2	31.1	43.6
	10	6.1	15.3	31.1	46.4	7.2	12.8	35.1	49.5	6.9	18.0	32.2	52.0
**control**	1	7.1	18.6	27.8	44.0	7.3	13.7	26.5	44.2	6.8	15.9	27.3	35.2
	2	6.8	19.1	24.9	43.5	6.9	14.2	28.3	47.1	7.2	16.8	26.7	40.2
	3	6.9	13.6	28.4	47.7	6.6	13.2	27.8	45.1	6.3	16.8	25.1	42.6
	4	6.5	17.8	26.9	45.8	8.3	12.2	26.7	46.8	7.9	17.2	25.4	42.5
	5	7.7	17.1	24.7	41.3	6.2	15.9	31.1	42.5	7.1	14.4	27.1	45.6
	6	5.9	16.4	31.5	45.1	6.2	15.4	26.8	47.4	7.2	13.0	21.9	40.5
	7	7.9	17.1	30.6	42.1	6.1	14.9	28.9	43.9	7.6	18.7	25.7	47.1
	8	7.5	17.4	30.6	44.2	6.6	13.1	29.9	48.1	6.8	15.4	28.4	45.7
	9	7.5	15.5	31.7	46.6	6.7	14.5	29.5	43.2	7.2	16.7	31.1	42.1
	10	7.6	16.3	31.5	43.5	7.3	14.8	27.5	43.2	8.1	17.6	33.0	42.0
		0wpv	4wpv	8wpv	12wpv	0wpv	4wpv	8wpv	12wpv	0wpv	4wpv	8wpv	12wpv
**vaccine**	**sum**	72.5	178.2	308.4	476.6	71.6	157.4	313.8	487.0	72.5	182.0	297.5	463.2
	**mean**	7.2	17.8	30.8	47.7	7.2	15.7	31.4	48.7	7.2	18.2	29.8	46.3
	**sd**	1.0	2.7	4.2	6.3	0.8	2.2	2.6	4.6	0.4	1.4	2.6	4.2
**control**	**sum**	71.4	168.9	288.6	443.8	68.0	141.9	283.0	451.5	72.2	162.5	271.7	423.5
	**mean**	7.1	16.9	28.9	44.4	6.8	14.2	28.3	45.2	7.2	16.3	27.2	42.4
	**sd**	0.6	1.6	2.7	2.0	0.6	1.6	2.7	2.0	0.5	1.6	3.1	3.4
